# Quality Evolution and Aroma Profile of Pointed Cabbage in Different Storage Regimes

**DOI:** 10.3389/fpls.2022.852817

**Published:** 2022-04-15

**Authors:** Maxime Janssens, Bert E. Verlinden, Maarten L. A. T. M. Hertog, Bart M. Nicolaï

**Affiliations:** ^1^Flanders Centre of Postharvest Technology, Leuven, Belgium; ^2^BIOSYST-MeBioS Postharvest Research Group, KU Leuven, Leuven, Belgium

**Keywords:** pointed cabbage, CA, long-term storage, quality, aroma, SPME-/TD-GC-MS

## Abstract

With its increasing popularity, the need for optimal storage conditions of pointed cabbages becomes more important to meet the year-round demand. Storage of the pointed varieties, however, is more difficult compared to the traditional, round varieties and is limited to a few weeks in normal air. Pointed cabbages are more susceptible to quality loss (shriveling, yellowing of leaves, weight loss, fungal, and bacterial infections) and tend to spoil much faster. In order to provide a year-round availability of the fresh product, storage under controlled atmosphere (CA) could offer a solution. In this study, pointed, white cabbage heads (*Brassica oleracea* var. capitata for. *alba* L. subv. *Conica* cv. ‘Caraflex’) were stored at 1°C from November 2018 to May 2019 under four different CA conditions (1 kPa O_2_ + 1.5 kPa CO_2_, 1 kPa O_2_ + 5 kPa CO_2_, 3 kPa O_2_ + 1.5 kPa CO_2_, and 3 kPa O_2_ + 5 kPa CO_2_), and compared to storage under normal air. Results showed that CA storage resulted in a prolonged storage life with a good quality retention for both texture and aroma. CA-stored cabbages showed less weight loss, shriveling, and yellowing. Internal quality parameters [color, soluble solids content (SSC)] were stable over the whole storage period for all objects. The aroma profiles of both the storage atmosphere and cabbage samples were impacted by storage duration. The aroma of cabbage juice was also affected by the storage regime. A clear separation was found for cabbage stored under CA compared to the reference group. From the CA-treatments studied, a combination of low oxygen (1 kPa O_2_) and elevated carbon dioxide levels (5 kPa CO_2_) showed the best results maintaining quality. Storage under CA resulted in a better resemblance to the aroma of freshly, harvested produce compared to cabbages stored in normal air.

## Introduction

White cabbage heads (*Brassica oleracea* L. var. capitata f. *alba*) are a popular crop typically grown in temperate climate regions (Gajewski et al., 2015) rich in vitamins, glucosinolates, and nutrient fibers (Suojala, 2003; Radovich, 2010). To ensure year-round availability, cabbage heads are stored up to 6 months at low temperature with high relative humidity (Wang, 2002). Its storability is affected by the developmental stage of the cabbages at harvest, with late crop cultivars being preferred for long-term storage (Cantwell and Suslow, 2001; Suojala, 2003). Despite their good storage potential, the main cost for marketing is preparing the cabbage heads for marketing by removing the outer leaves prone to postharvest shriveling, senescence, and fungal and bacterial infections (Menniti et al., 1997). Losses due to trimming of the cabbage heads for cosmetic display may mount up to a total weight reduction of 20% (Munhuweyi et al., 2016).

While storage under controlled atmosphere (CA) is widely used for fruit storage, it is less adopted for vegetable storage due to the high diversity of plant materials that all can react differently under the same CA conditions (Raghavan et al., 1984). The main effect of low temperature, retarding the loss of quality (Suojala, 2003) and lowering the respiration rate (Ratti et al., 1996) can, however, be further optimized by additionally controlling the gas conditions in the cool room. Multiple reports have shown that storage of cabbage heads in CA results in better color retention, less water loss and leaf shriveling, a decrease in fungal infections, higher retention of concentrations of chlorophyll and total sugar content and lower respiration rates (Raghavan et al., 1984; Menniti et al., 1997; Wang, 2002; Gajewski et al., 2015). Information on the effect of CA on pointed varieties, however, is limited. Pointed cabbages are mainly grown in western Europe where they, given their limited storability, usually target the fresh market. Due to their smaller size and sweeter taste, the pointed varieties are gaining popularity increasing their demand year-round. Gajewski et al. (2015) found that CA storage under 3 kPa O_2_ and 5 kPa CO_2_ combined with low temperature decreases quality loss over a 3 month storage period. CA storage of pointed cabbage is currently successfully applied by some innovative Dutch growers, prolonging the storage period for several months by lowering O_2_ concentrations down to 1 kPa.

Since consumer acceptance of fresh cabbage is, besides to textural attributes, highly related to its aromatic attributes, it is important to include cabbage aroma when studying its quality evolution during storage. Fresh cabbage flavor can be sweet, fruity, and rich, however, it can also be bitter, musty, and flat. The bitterness and pungency of the cabbage flavor is reported to be influenced mainly by the concentrations of sulfides, glucosinolates and their related hydrolysis products (Radovich, 2010). Storage in a CA regime can help preserve a good aroma, but is not without risks. Multiple research reports state the development of off-odors and mustiness when O_2_ levels drop below a certain threshold or when CO_2_ levels are too high during storage (Menniti et al., 1997; Radovich, 2010).

The aim of this study is to evaluate if storage under CA prolongs the storage duration of pointed cabbage heads while maintaining good quality, and if results show that CA storage is beneficial, to study if the volatile profile can be linked to quality in order to use non-destructive aroma measurements to monitor quality during the storage season. Therefore, in this study, fresh white pointed cabbage heads were stored for 6 months to study the effect of CA storage on product quality covering both textural and aromatic aspects. The influence of low O_2_ levels (1–3 kPa) and increased CO_2_ levels (1.5–5 kPa) in the storage atmosphere was studied and compared to traditional cold storage under normal air. Though volatile aroma compounds are produced inside the tissue, depending on their solubility, they equilibrate to certain levels with the headspace surrounding the produce. To obtain a complete picture, the aroma volatiles were measured both at the level of the tissue (by analyzing juice samples using SPME-GC-MS) and that of the headspace (using TD-GC-MS). In order to maintain quality, it is desirable to keep both the internal (juice) and external (headspace) aroma stable and close to the aroma of freshly harvested produce.

## Materials and Methods

### Plant Material

White, pointed cabbage heads (*Brassica oleracea* var. capitata for. *alba* L. subv. *Conica* cv. ‘Caraflex’) were grown commercially and harvested on November 26, 2018 in Poederlee (51.25510°N, 4.84442°E), Belgium. The fresh produce was transported to the laboratory where it was randomized. In accordance with commercial practice, the produce was first stored under regular air at 1°C for 1 week before being exposed to the different storage regimes for long-term storage experiments.

### Storage Facilities

The cabbage heads were stored at 1°C in containers (0.715 m × 0.515 m × 0.965 m) made of 5 mm thick polypropylene closed air-tight with a lid in a water lock. Each container contained five EPS medium crates with seven cabbages each, 35 cabbage heads in total. On December 4, 2018, all containers were connected to a CA controller unit equipped with a fluorescence based optic oxygen sensor (SST Sensing Ltd., Coatbridge, United Kingdom), a non-dispersive infrared based carbon dioxide sensor (GE-Telaire T6615-50KF 50,000 ppm, TELAIRE, United States) and a digital pressure sensor (MEAS MS5611-01BA03, MEAS, Switzerland). The CA unit measured the gas concentrations in the containers every 2 h followed by a series of injections to make corrections as needed. The applied CA conditions were 1 kPa O_2_ + 1.5 kPa CO_2_, 1 kPa O_2_ + 5 kPa CO_2_, 3 kPa O_2_ + 1.5 kPa CO_2_, and 3 kPa O_2_ + 5 kPa CO_2_. As a reference treatment, containers were flushed continuously at a flow rate of 5 L min^–1^ with normal air. Per storage treatment, 3 replicate containers, each containing 35 cabbage heads, were studied.

### Quality Measurements

Product quality was evaluated at harvest (November 2018), halfway (February 2019), and at the end of the storage season (May 2019). These timepoints will further be indicated as harvest, 94 days after harvest (“dah”) and 163 dah, respectively. At each time point, measurements were done on the day of opening the container (shelf life zero days, “SL0”) and after 7 days on the shelf at 7°C (“SL7”). Per assessment, nine replicate cabbage heads were measured (three cabbage heads × three containers per treatment).

#### Weight Loss

The weight of the crates was measured using a Defender 3000 balance with a measurement range of 0.04–60 kg (OHAUS Europe GmbH, Nänikon, Switzerland). This measurement was done at each quality assessment to determine water loss during storage. For each individual cabbage head, the weight was measured with a CP4201 balance with a range of 0.1–4200 g (Sartorius AG, Göttingen, Germany). The outer leaves were peeled, i.e., the loose, shriveled, discolored, and infected leaves were removed in order to obtain a tight, green and saleable crop. After peeling, the weight of the cabbages was measured again. A picture of the intact cabbage heads was taken before and after pealing, for further analysis.

#### Leaf Scoring and Color Measurement

Preliminary studies showed high variability for color measurements on the outer leaves of cabbage heads due to the non-homogenous discoloration of the leaves (data not shown). In order to evaluate the external color of the cabbage heads, pictures taken of the cabbage heads before pealing of the outer leaves (paragraph in section “Weight Loss”) were used to give the cabbage heads a leaf score. A cabbage head that has fresh, fully green leaves received score “0.” When the leaf edges showed signs of yellowing, score “1” was given, cabbage heads with outer leaves with yellow parts received score “2.” From the moment that brown parts were present, the cabbage head received score “3.” In Supplementary Figure 1, a reference scale is presented.

Additional to the leaf scoring, a color measurement of the internal parts was carried out. Preliminary studies showed that cabbage heads can show internal browning due to O_2_ deficiencies, while maintaining a good external look. For this internal color measurements, the cabbage heads were cut in half from top to stem end. The color of the cut surface was measured five times around the stem using a CM-2600d spectrophotometer (Konica Minolta Sensing Ltd., Singapore), avoiding the main vein. The values were averaged to obtain the mean value for that cabbage head.

#### Soluble Solids Content

Half a cabbage head was turned into juice using a juice extractor (Philips, Netherlands). The soluble solids content (SSC) of the juice was measured using a refractometer Palette PR-101α (Atago Ltd., Tokyo, Japan) with a range of 0–45%.

#### Statistical Analysis

Data from the quality assessments were statistically analyzed using JMP PRO 11 (SAS, United States). A full-factorial ANOVA analysis (*p* < 0.05) was carried out on the quality parameters based on the following factors: the different storage conditions (five levels), storage time (three levels), shelf life (two levels), and their interaction effect. Subsequently, differences between treatment level means were detected by a pairwise comparison using the Tukey HSD test (*p* < 0.05). The heteroscedasticity of the weight loss data was normalized using a logarithmic transformation before the ANOVA analysis was carried out. An ordinal logistic regression was carried out on the leaf scoring data to study the effect of time, shelf life, O_2_, and CO_2_. Based on Akaike’s criterium, an interaction term between shelf life and CO_2_ was included in the statistical analysis.

### Aroma Measurements

#### Measurement of the Volatile Composition of the Headspace Surrounding the Produce in the Storage Containers

The aroma volatile composition of the headspace surrounding the cabbage heads in the storage containers was measured in the beginning of the CA storage (December), middle (February), and end (May) of the storage season. The authors will refer to these measurements as “start storage,” “86 days after start storage protocols (das),” and “155 das,” respectively. The samples taken from the containers at start are grouped into one group, since all of them had the same pre-treatment. Prior to sampling, the headspace surrounding the cabbage heads in the storage containers, was flushed for 24 h with a gas mixture according to its storage treatment to refresh the containers atmosphere. This way, a comparable aroma volatile composition of the headspace surrounding the cabbage heads was generated during a subsequent fixed accumulation period of 24 h in each container during which the containers were closed air-tight. An air sample of the storage container was taken in a 5 L Tedlar bag (MediSense, Netherlands). At each time point, an air sample was taken from the storage containers to analyze the volatile composition of the headspace surrounding the cabbage heads. This resulted in seven, two, and three biological replicates per treatment at start, 86 and 155 das, respectively.

An air sampling pump (Gilian GilAir Plus, Sensidyne, LP, United States) drew 1000 mL of the gaseous sample from the Tedlar bags at a flow rate of 100 mL min^–1^ over a glass tube filled with glass beads held on ice onto multisorbent thermal desorption (TD) tubes. The tube with glass beads was added in series to reduce the moisture content of the air samples. Multi-bed adsorbent tubes were used containing a series of three different adsorbents beds: Carbopack B, Carbopack X, and Carbosieve-III (GERSTEL GmbH & Co. KG, Germany).

A gas chromatograph (GC, 7890A, Agilent Technologies, Santa Clara, CA, United States) coupled to a mass spectrometer (MS, 5975C, Agilent Technologies, Santa Clara, CA, United States) was equipped with a thermal desorption unit (TDU, GERSTEL GmbH & Co. KG, Mülheim an der Ruhr, Germany) and cooled injection system (CIS) (CIS 4, GERSTEL GmbH & Co. KG, Mülheim an der Ruhr, Germany), cooled with liquid nitrogen. The TD tubes were placed in the TDU and dry purged for 5 min at 10°C to remove excess water. The adsorption tubes were then thermally desorbed using a splitless program going from 10 to 270°C (hold 4 min) at a rate of 100°C min^–1^ in order to release all adsorbed volatiles. These compounds were then cryo-focused in the CIS equipped with a notched inner glass liner filled with Carbotrap B. A CIS heating program from 50 to 250°C with a rate of 12°C min^–1^ and a 5 min hold injected the analytes into the GC with helium as carrier gas with a flow of 50 mL min^–1^. With an oven temperature rising from 35 to 170°C at a 10°C min^–1^ rate (hold 0 min) and from 170 to 250°C at 20°C min^–1^ (hold 5 min) and a constant carrier flow of 1 mL min^–1^, the analytes were separated on a 25 m long Pora Bond Q column (CP7348PT, Agilent J&W GC columns). The column had a 0.25 mm diameter and 3 μm film thickness. The volatiles were then detected in the MS-detector using a full scan, 12–350 amu at a scan rate of 4.17 Hz.

#### Aroma Measurement of Cabbage Juice

For each storage regime, at each time point (i.e., harvest, 94 dah and 163 dah) and for each shelf-life (SL0, SL7), nine biological replicates (i.e., cabbage heads) where studied using SPME-GC-MS. Of each cabbage, half of the head – cut from top to stem – was turned into juice using a juice extractor (Philips, Netherlands). A Falcon^®^ tube was filled with a mixture of cabbage juice (6 g) and saturated NaCl (6 g). The salt is added to stabilize the cabbage juice sample and to obtain an effective extraction onto the fiber thanks to the “salting out” effect (Belton, 1937; Kataoka et al., 2000). All samples were frozen in liquid nitrogen and stored at −80°C until analysis. On the day of analysis, the sample was defrosted and a 10 g specimen was transferred into a glass vial (20 mL Headspace Vial, 75.5 × 22.5 mm clear glass, BIPP, Macherey-Nagel GmbH), which was flushed for 10 s with clean nitrogen gas and sealed using a cap (Crimp closure, N20, alu., Center hole, Silicone blue transp./PTFE colorl., 3.0 mm, Macherey-Nagel GmbH). The vial was placed on a heated tray (35°C) for an incubation and extraction of 30 min each. During the extraction, a 65 μm CAR-PDMS-DVB (Supelco Inc., United States) fiber was placed in the surrounding headspace of the sample (i.e., through the septum into the vial) by a GERSTEL MultiPurpose Sampler 2 (GERSTEL GmbH & Co. KG, Germany).

After extraction, the aroma compounds were thermally desorbed into the injector (heated at 250°C) of the Agilent 6890N gas chromatograph (GC, Agilent Technologies, Santa Clara, CA, United States), equipped with a SPME liner (0.75 mm internal diameter, Supelco Inc., United States). The splitless injection was performed with a purge flow of 50 mL min^–1^ for 0.75 min and the TD of the fiber took 6 min. The volatiles were separated on an Optima-5MS column (30 m × 0.25 mm internal diameter × 0.25 μm film thickness) (Agilent J&W, Agilent Technologies, United States) with an oven temperature rising from 35 to 150°C at a rate of 4°C (hold 0 min) and to 240°C at a rate of 50°C min^–1^ (hold 5 min) with a constant flow of 1.2 mL min^–1^ using helium as a carrier gas. The volatiles were then detected with an Agilent 5975N MSD Mass Selective Detector (MS, Agilent Technologies, Santa Clara, CA, United States) recording mass spectra in the 15–350 m/z range at a scanning speed of 7.76 scans per second.

#### Analysis of Chromatograms and Compound Identification

The chromatograms and corresponding mass spectra obtained with TD- and SPME-GC-MS were deconvoluted and analyzed using MSD Chemstation (Agilent Technologies, United States) and the automated mass spectral deconvolution and identification software Unknowns (Masshunter Workstation software, version B.09.00, Agilent Technologies). The NIST 14 mass spectral library [National Institute of Standards and Technology (NIST), United States] was used for identification of the aroma volatiles. Compounds obviously coming from the instrument (e.g., siloxanes from septa or liner) or the experimental setup (i.e., background from the storage containers), as determined based on blank measurements, were omitted from the analyses. For the analysis of the aroma data, the abundancy of each compound of interest was expressed relative to the total aroma.

#### Multivariate Data Analysis

The multivariate aroma data was analyzed in The Unscrambler^®^ X software (Version 10.5.1, 2018, CAMO Software AS., Oslo, Norway). Data was mean centered and variables were weighted by the standard deviation. A principal component analysis (PCA) was carried out first, followed by a partial least squares (PLS) regression. In the PLS analysis, the volatiles were defined as the *X*-variables, and the *Y*-variables were, respectively, O_2_ or CO_2_ concentration and time. For SPME-GC-MS, an additional *Y*-variable “shelf life” was included. Jack-knifing was used to determine the statistically significant (*p* < 0.05) volatiles.

## Results

### Quality Evolution During Storage

**FIGURE 1:**
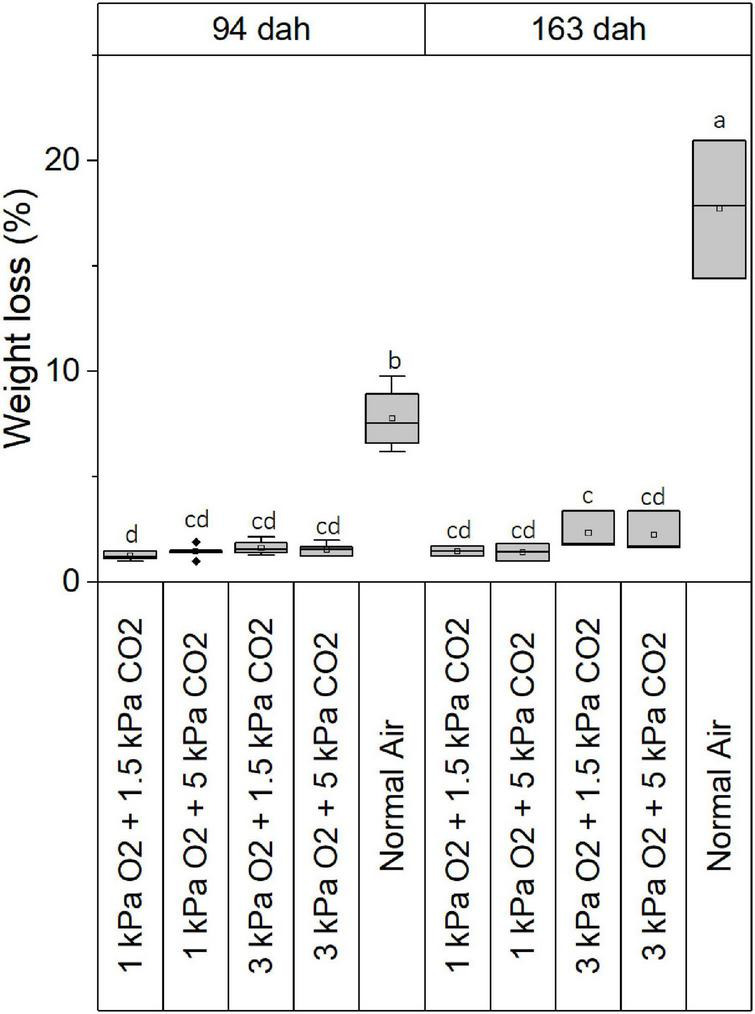
Boxplots representing the weight loss of ‘Caraflex’ cabbages stored under different storage conditions 94 dah and 163 dah. Statistical differences (*p* < 0.05) between treatments are indicated by different letters.

Figure 1 presents boxplots showing the weight loss of ‘Caraflex’ cabbages 94 dah and 163 dah for all four CA storage regimes. For the control group (i.e., cabbages stored in normal air) a weight loss of 8% (94 dah) to 18% (163 dah) was found which was at both time points much higher compared to all CA stored cabbages which showed losses below 2.5%. Within the different CA treatments, no significant differences were found.

**FIGURE 2:**
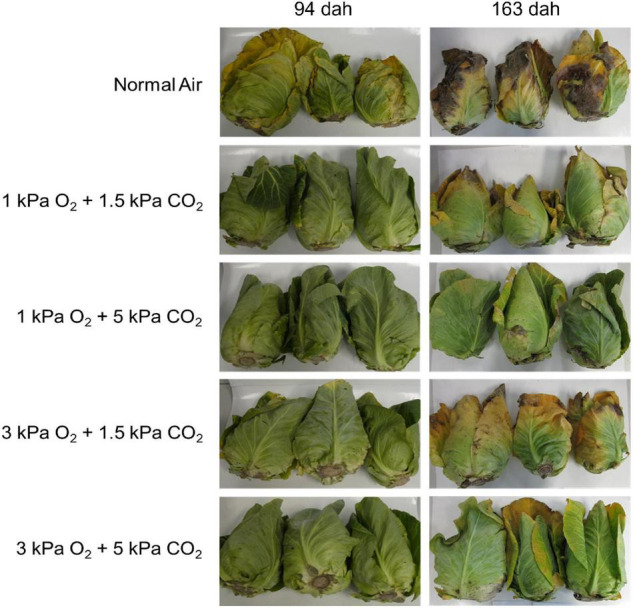
Cabbage heads 94 dah and 163 dah in different storage conditions on the day of opening the storage containers (shelf life zero days, “SL0”).

**FIGURE 3:**
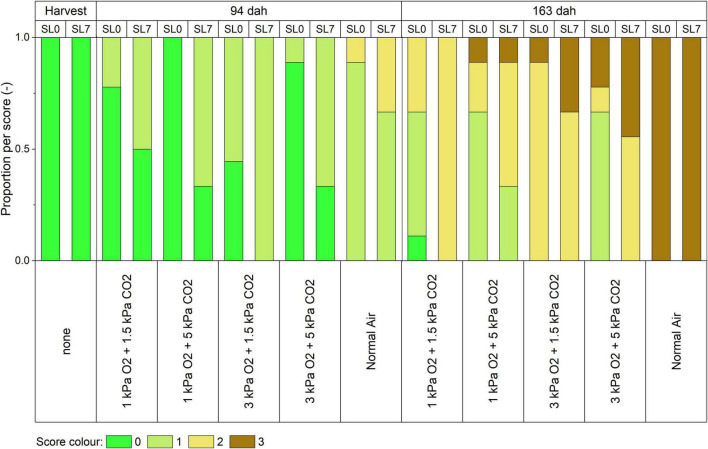
External quality evolution of pointed cabbages during storage and shelf life. The outer leaves of nine biological replicates were scored at harvest, 94 dah and 163 dah in different atmospheric conditions on the day of opening the containers (shelf life zero days, “SL0”) and after 7 days on the shelf at 7°C (“SL7”). The leaves obtained a score according to the scale presented in Supplementary Figure 1. The *p*-values per factor of the ordinal logistic regression are presented in Table 1.

Figure 2 shows representative pictures of the outer appearance of cabbages 94 dah and 163 dah after storage in the different storage conditions. Cabbages stored in normal air showed a faster decay, with yellowing and shriveling already started 94 dah and brown, rotten leaves appearing 163 dah. The different CA treatments showed a better quality retention, with still freshly looking product 94 dah. For 163 dah, the CA treatments all showed some yellowing; however, the CA treatment with 1 kPa O_2_ + 5 kPa CO_2_ showed the most fresh, green leaves, whilst the CA treatment with 3 kPa O_2_ + 1.5 kPa CO_2_ showed the most yellowing. This quality was objectively scored on a scale (Supplementary Figure 1) from green “0” to brown “3” in Figure 3. Halfway the storage season (94 dah) all CA regimes maintained green, healthy leaves, with limited yellowing and shriveling on the day of opening the container (“SL0”). After a week at 7°C, yellowing of the leaves became more prominent. However, 163 dah all CA-stored cabbages showed (partial) yellowing at SL0 and in some cases even brown spots at SL7.

An ordinal logistic regression (Table 1) shows that both storage time (*p* < 0.0001) and shelf life (*p* < 0.0001), and the concentrations of both O_2_ (*p* < 0.0001) and CO_2_ (*p* < 0.0344) have a significant role on the obtained leaf score. A certain loss in quality will occur with increased storage duration or days on the shelf at 7°C. However, the rate of degradation can be slowed down by decreasing the level of O_2_ and increasing the level of CO_2_ during storage. It appears that the effect of O_2_ is bigger compared to the effect of CO_2_. The interaction effect of CO_2_× shelf life is nearly, however, not significant (*p* = 0.0574). Although, according to Akaike’s criteria, it is beneficial to include this interaction term into the model. It appears that the positive effect of increased CO_2_ is lost with increased shelf life, however, based on the *p* > 0.05, this was not confirmed in this study.

**TABLE 1:** Results of the ordinal logistic regression carried out on the leaf score data presented in Figure 3.

	*p*-Value
Time	<0.0001*
O_2_	<0.0001*
CO_2_	0.0344*
Shelf life	<0.0001*
CO_2_ × shelf life	0.0574

If p < 0.05 (indicated by *), the factor has a significant effect on the model describing the leaf score of the outer leaves according to the effect likelihood ratio tests.

Despite the differences in outer appearance, 94 dah, cabbages of all five treatments were suited for marketing after trimming. A weight loss of about 20% was noted due to this process for all storage treatments. At 163 dah, more extensive trimming was necessary (about 26%). For the control treatment, however, trimming was not sufficient to obtain a nice, saleable product since the inner leaves showed yellowing and decay as well.

**FIGURE 4:**
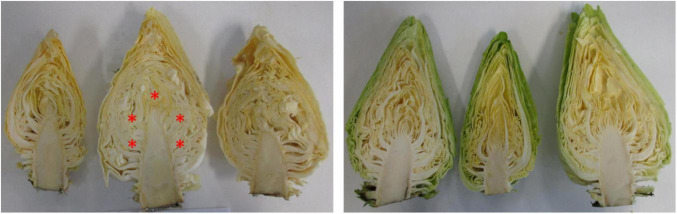
Internal quality development of pointed cabbages stored for 6 months under normal air (left) or 1 kPa O_2_ + 5 kPa CO_2_ (right). Every cabbage was measured five times on the center parts avoiding the main vein as indicated by the red asterisk (*). The values were averaged to obtain the mean value for each cabbage head.

Internal quality was described by the internal color of cut cabbage surface and SSC of the cabbage juice. In Table 2, the values for the perceptual lightness (L*), how the color is perceived (Hue) and the SSC were presented. Despite the clear discoloration of the outer leaves, no statistical difference was found between different treatments for the L* and hue values measured on the cut surface of the cabbages. In Figure 4 this is confirmed since no obvious visual differences can be seen. The SCC (Table 2) appeared to be stable during long-term CA storage. For the control treatment, a small increase was found. This increase was significant for SL0 but disappeared for SL7 and was too small to be noticeable during consumption of the product.

**TABLE 2:** Color values measured on the inner surface and soluble solids content (SSC) of juice of white conical cabbages at harvest, 94 dah and 163 dah after storage in different storage regimes.

			L*	Hue	SSC (%)
**Harvest**					
		SL0	74 ± 4 (a)	93 ± 1 (a)	9.5 ± 0.4 (d)
		SL7	72 ± 6 (a)	92.0 ± 0.9 (ab)	10.1 ± 0.3 (bcd)
**94 days after harvest**					
	Normal air	SL0	78 ± 5 (a)	91 ± 1 (abc)	11.0 ± 0.8 (ab)
		SL7	75 ± 4 (a)	92 ± 1 (abc)	10.7 ± 0.5 (abc)
	1 kPa O_2_ + 1.5 kPa CO_2_	SL0	76 ± 6 (a)	91 ± 1 (abc)	*9.5 ± 0.6 (d)*
		SL7	74 ± 6 (a)	91 ± 2 (abc)	9.7 ± 0.2 (cd)
	1 kPa O_2_ + 5 kPa CO_2_	SL0	73 ± 6 (a)	91 ± 1 (abc)	9.7 ± 0.4 (cd)
		SL7	74 ± 8 (a)	91 ± 1 (abc)	9.7 ± 0.9 (cd)
	3 kPa O_2_ + 1.5 kPa CO_2_	SL0	75 ± 7 (a)	92 ± 1 (abc)	9.8 ± 0.5 (cd)
		SL7	71 ± 7 (a)	91 ± 1 (abc)	9.8 ± 0.5 (cd)
	3 kPa O_2_ + 5 kPa CO_2_	SL0	74 ± 7 (a)	91.1 ± 0.8 (abc)	9.8 ± 0.6 (cd)
		SL7	74 ± 4 (a)	91 ± 1 (abc)	9.9 ± 0.5 (bcd)
**163 days after harvest**					
	Normal air	SL0	80 ± 2 (a)	91.1 ± 0.8 (abc)	11 ± 1 (a)
		SL7	79 ± 4 (a)	91 ± 0.5 (bc)	10 ± 1 (bcd)
	1 kPa O_2_ + 1.5 kPa CO_2_	SL0	77 ± 7 (a)	91 ± 1 (bc)	*9.6 ± 0.4 (cd)*
		SL7	79 ± 5 (a)	91 ± 1 (abc)	9.6 ± 0.4 (cd)
	1 kPa O_2_ + 5 kPa CO_2_	SL0	75 ± 5 (a)	90 ± 1 (c)	9.2 ± 0.4 (d)
		SL7	80 ± 5 (a)	90.7 ± 0.8 (bc)	9.6 ± 0.5 (cd)
	3 kPa O_2_ + 1.5 kPa CO_2_	SL0	78 ± 4 (a)	91 ± 1 (abc)	9.8 ± 0.7 (cd)
		SL7	78 ± 5 (a)	90 ± 1 (c)	9.9 ± 0.5 (cd)
	3 kPa O_2_ + 5 kPa CO_2_	SL0	75 ± 5 (a)	90.6 ± 0.9 (bc)	9.7 ± 0.8 (cd)
		SL7	80 ± 6 (a)	91 ± 1 (abc)	9.3 ± 0.6 (d)

*Per parameter, treatments with a different letter are significantly different from each other (Tukey HSD test, p < 0.05).*

### Aroma Measurements

#### Headspace Aroma (TD-GC-MS)

**FIGURE 5:**
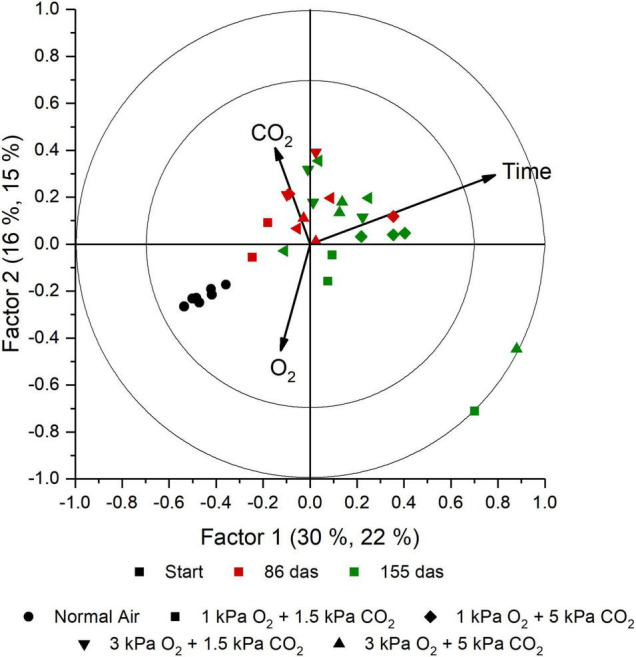
Score plot obtained with the PLS analysis of the aroma measurement of the container headspace of storage containers using TD-GC-MS. Between the brackets per factor, the first percentage indicates the percentage of which part of the aroma variables are represented in the projection of this dimension, while the second percentage indicates to what extent the response is explained by this. The different objects are color- and shape-coded based on time and treatment, respectively, as indicated in the legend. Start includes measurements of seven (i.e., biological replicate) containers when the different storage regimes are started. For 86 and 155 days after storage started, two and three biological replicates were measured per storage conditions.

**FIGURE 6:**
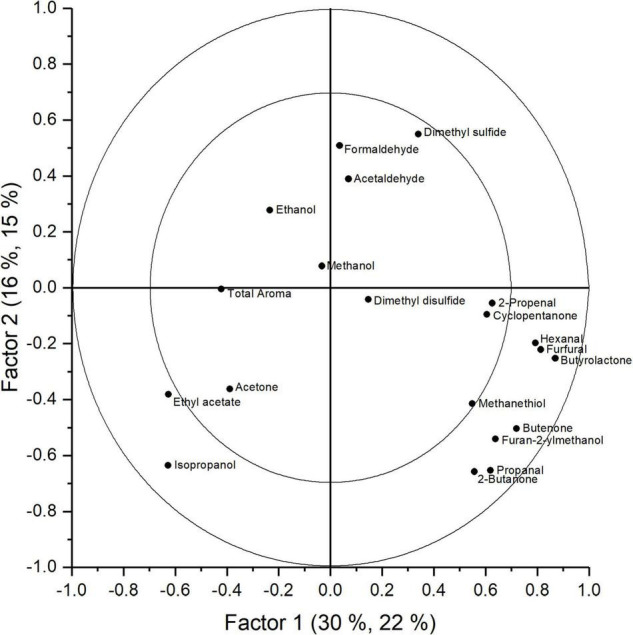
Loadings plot obtained with the PLS analysis of the aroma measurement of the headspace in the storage containers analyzed with TD-GC-MS.

From the PCA analysis (data not shown) we found no clear distinction between groups. The main discriminative factor in the model was time, showing a change in aroma with increasing storage duration. The model was, however, not able to discriminate between different levels of O_2_ and CO_2_. In the PLS analysis, these findings were confirmed. A PLS model with seven factors was obtained. For time, the explained variance reaches 93.5% for the full model with seven factors and a cross-validation of 76.7% (Supplementary Figure 2). For O_2_ and CO_2_, no meaningful models could be obtained as the cross-validation completely failed. The model appears to have difficulty to distinguish groups based on the O_2_ and CO_2_ levels. In Figure 5, the score plot of the two first factors is presented. The colors represent the storage duration and a shift in aroma profile is seen with increased storage duration. The first two factors explain a 37% of the total *Y* variance (including time, O_2_, and CO_2_); however, the first two factors describe 70.5% of the time effect (Supplementary Figure 2). The model is unable to distinguish samples based on the storage regime (symbols). The corresponding loading plot is presented in Figure 6. Per aroma compound, the *p*-values for O_2_, CO_2_, and time are presented (Table 3). From the Jack-knifing, it was found that seven aroma compounds determined the change in aroma profile due to the storage duration. The change in the relative abundancies of these seven aroma compounds is shown in Supplementary Figure 3. At harvest, higher levels of acetone, isopropanol and ethyl acetate were found compared to later in the season. With increasing storage duration, the level of the well-known off-odor dimethyl disulfide (Akpolat and Barringer, 2015; Rajkumar et al., 2017) showed an increasing trend. This trend was not significantly robust, however, the compound showed to have a significant role describing the time effect in de PLS model (Table 3). Furfural and butyrolactone also had a significant role in describing the time effect and showed an increase during storage. Formaldehyde did not change significantly with time. Although that the model validation did not withstand describing the effect of either O_2_ or CO_2_, formaldehyde, dimethyl disulfide and furan-2-ylmethanol show a significant *p*-value for the Jack-knifing, indicating that these compounds might be important parameters in future. The change of the relative abundancies of these compounds are shown in Supplementary Figures 4, 5, respectively. In Supplementary Table 1 the relative values of all aroma compounds of interest are shown per treatment and storage time.

**TABLE 3:** List of aroma compounds found in the headspace surrounding the cabbages in storage (TD-GC-MS).

	O_2_	CO_2_	Time
Formaldehyde	<0.05*	<0.05*	<0.05*
Methanol	0.54	0.48	0.59
Acetaldehyde	0.50	0.93	0.16
Methanethiol	0.38	0.34	0.07
Ethanol	0.92	0.79	0.14
2-Propenal	0.30	0.86	0.63
Propanal	0.26	0.12	0.68
Acetone	0.99	0.99	<0.05*
Isopropanol	0.73	0.98	<0.05*
Dimethyl sulfide	0.06	0.07	0.10
Butenone	0.25	0.42	0.11
2-Butanone	0.43	0.17	0.29
Ethyl acetate	0.49	0.38	<0.05*
Furfural	0.32	0.40	<0.05*
Dimethyl disulfide	<0.05*	<0.05*	<0.05*
Cyclopentanone	0.81	0.88	0.85
Furan-2-ylmethanol	0.11	<0.05*	0.67
Hexanal	0.26	0.73	0.12
Butyrolactone	0.15	0.23	<0.05*
Total aroma	0.67	0.93	0.93

For each Y-variable (O_2_, CO_2_, and storage time), the p-values from the Jack-knifing are presented. If p < 0.05 (indicated by *), the aroma compound has a significant effect on the model variable.

#### Cabbage Juice Aroma (SPME-GC-MS)

**FIGURE 7:**
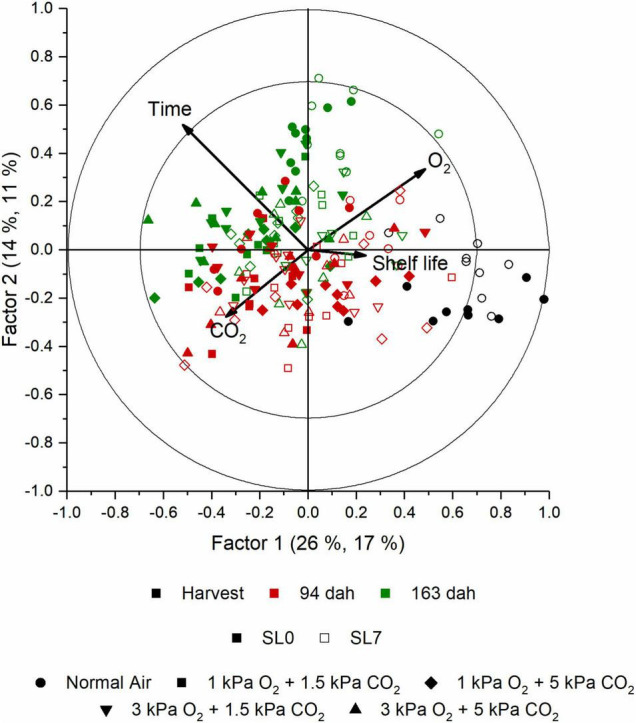
Score plot of factor 1 and 2 obtained with the PLS analysis of the aroma measurement of the cabbage juice using SPME GC-MS. Between the brackets per factor, the first percentage indicates the percentage of which part of the aroma variables are represented in the projection of this dimension, while the second percentage indicates to what extent the response is explained by this. The different objects are color- and shape-coded based on time and treatment, respectively, as indicated in the legend. At each time point, shelf life and storage treatment, nine biological replicates were measured.

**FIGURE 8:**
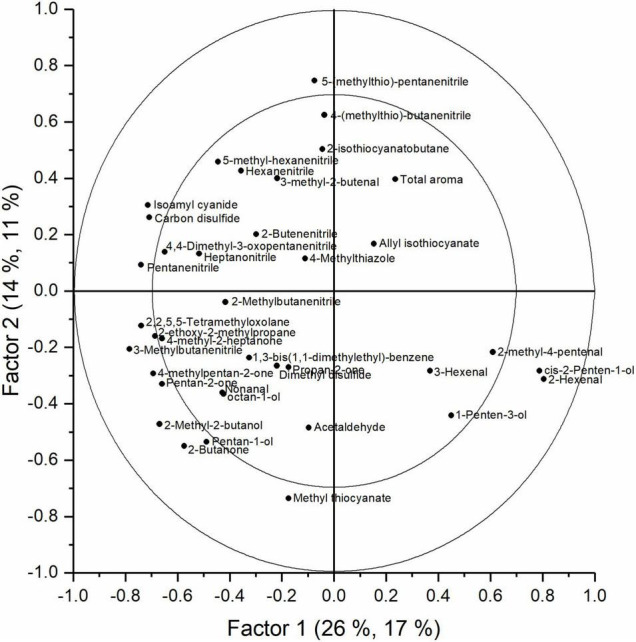
Loadings plot of factor 1 and 2 obtained with the PLS analysis of the aroma measurement of cabbage juices analyzed with SPME-GC-MS.

**FIGURE 9:**
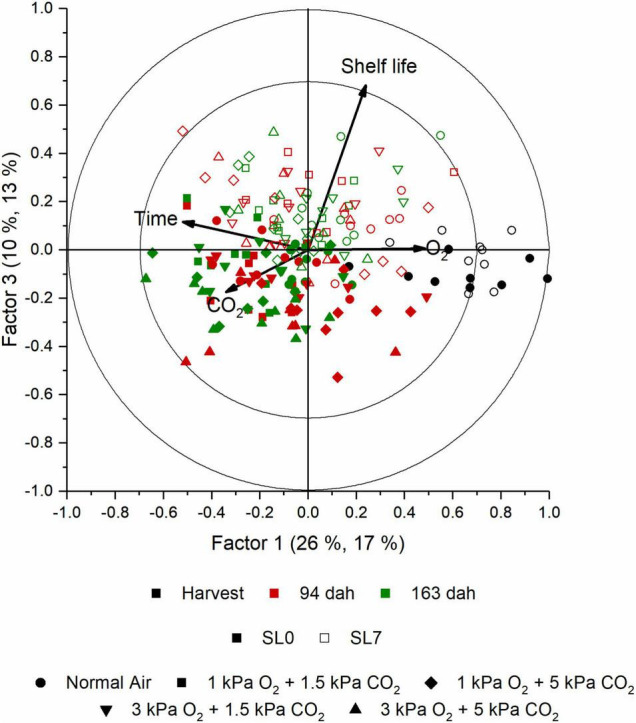
Score plot of factor 1 and 3 obtained with the PLS analysis of the aroma measurement of the cabbage juice using SPME GC-MS. Between the brackets per factor, the first percentage indicates the percentage of which part of the aroma variables are represented in the projection of this dimension, while the second percentage indicates to what extent the response is explained by this. The different objects are color- and shape-coded based on time and treatment, respectively, as indicated in the legend. At each time point, shelf life and storage treatment, nine biological replicates were measured.

**FIGURE 10:**
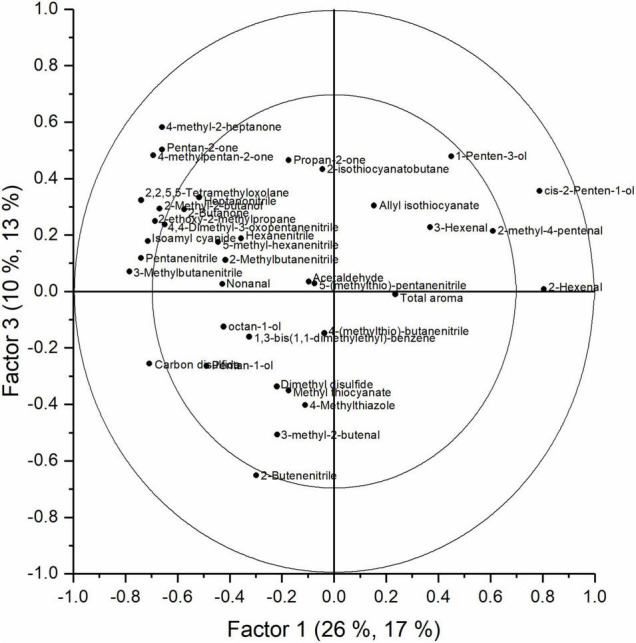
Loadings plot of factor 1 and 3 obtained with the PLS analysis of the aroma measurement of cabbage juices analyzed with SPME-GC-MS.

The aroma of cabbage juice was analyzed using SPME-GC-MS. The PCA analysis of the juices (data not shown) indicated that there is a change in aroma profile with an increased storage duration and shelf life. Besides time, O_2_, and CO_2_ levels were important discriminative factors too. In Supplementary Figure 6, the explained *Y* variances per factor are presented. The explained variance for a model with seven factors was equal to 78.5, 63.9, 62.4, and 46.0% for time, shelf life, O_2_, and CO_2_, respectively; the corresponding cross-validation explained variance was 72.5, 54.2, 52.2, and 35.3%. Time appeared to be the main discriminative factor, but both shelf life and storage regime also influenced the aroma profile of the cabbage juices. In Figure 7, the score plot of a PLS analysis shows how the aroma changed during the storage season for all objects. In this plot, the first two factors are shown. The first two factors explained 28% of the total *Y* variance (including time, shelf life, O_2_, and CO_2_); however, the first two factors accounted for 55.8, 5.7, 34.7, and 18.5% of the time, shelf life, O_2_, and CO_2_ effect, respectively (Supplementary Figure 6). In the score plot, the effect of time was clearly visible since the samples taken at harvest were separated from the other samples. The samples from the middle and the end of the season were less clearly separated. The corresponding loading plot is presented in Figure 8. In Figure 9, the score plot of the first and third factors are presented. These factors explain 30% of the total *Y* variance (including time, shelf life, O_2_, and CO_2_); however, the effect of shelf life (52% explained variance) is clearly present in factor 3 (Supplementary Figure 6). The corresponding loading plot is presented in Figure 10. The corresponding list of volatiles and their *p*-values that indicate their importance to the model are tabulated in Table 4. The aroma is characterized by lots of sulfides, nitriles, and (iso)thiocyanates, which are possible hydrolysis products from glucosinolates (McNaughton and Marks, 2003; Melrose, 2019). According to the results of the Jack-knifing, different aroma compounds were found to contribute to explain the variability of the different *Y* variables. The change in the relative abundancies of these aroma compounds is shown in Supplementary Figures 7–10. These aroma compounds all contribute to the effect of either time, shelf life, O_2_, and CO_2_. The aroma of the juice at harvest was characterized by 2-hexenal and 1-penten-3-ol which are linked to green, fresh notes (Burdock, 2010). Both showed higher concentrations when O_2_ levels are higher. Different sulphureous compounds were correlated with the different *Y* variables. The level of carbon disulfide was increased during storage under high O_2_ and low CO_2_ levels and also increased during storage. Nitrile levels also changed during storage under CA. In general, higher levels of nitriles were found at the end of the storage, but no clear pattern is found for the effect of O_2_ and CO_2_ on their concentration. In Supplementary Table 2 the relative values of all aroma compounds considered are shown per treatment and storage time.

**TABLE 4:** List of aroma compounds found in the headspace surrounding the cabbages in storage (SPME GC-MS).

	O_2_	CO_2_	Time	Shelf life
Acetaldehyde	0.06	0.14	<0.05*	0.88
Propan-2-one	0.45	0.44	0.70	<0.05*
Carbon disulfide	<0.05*	<0.05*	<0.05*	0.36
2-Butanone	0.12	0.22	<0.05*	0.30
2-Ethoxy-2-methylpropane	0.74	0.94	0.68	0.15
2-Methyl-2-butanol	0.63	0.66	0.34	0.35
2-Butenenitrile	<0.05*	<0.05*	<0.05*	<0.05*
1-Penten-3-ol	<0.05*	<0.05*	0.05	<0.05*
Pentan-2-one	0.26	0.25	0.24	<0.05*
Methyl thiocyanate	0.19	0.16	<0.05*	<0.05*
2-Methylbutanenitrile	0.99	0.53	0.73	0.06
3-Methylbutanenitrile	0.70	0.85	0.81	<0.05*
4-Methylpentan-2-one	0.59	0.62	0.61	<0.05*
Dimethyl disulfide	0.68	0.46	0.28	<0.05*
2,2,5,5-Tetramethyloxolane	0.08	0.22	0.06	0.63
3-Methyl-2-butenal	1.00	0.58	0.33	<0.05*
Pentan-1-ol	0.44	0.29	0.10	<0.05*
*cis*-2-Penten-1-ol	0.63	0.85	0.34	<0.05*
Pentanenitrile	0.44	0.69	0.09	0.39
3-Hexenal	<0.05*	<0.05*	<0.05*	0.11
2-Methyl-4-pentenal	0.83	0.41	0.09	0.73
4,4-Dimethyl-3-oxopentanenitrile	0.50	0.22	0.71	0.89
Isoamyl cyanide	<0.05*	<0.05*	0.68	0.20
2-Hexenal	<0.05*	<0.05*	<0.05*	0.23
Allyl isothiocyanate	0.25	0.45	0.13	<0.05*
Hexanenitrile	0.86	0.45	0.07	0.19
2-Isothiocyanatobutane	0.51	0.87	<0.05*	<0.05*
4-Methyl-2-heptanone	<0.05*	<0.05*	<0.05*	<0.05*
5-Methyl-hexanenitrile	0.52	0.09	0.64	<0.05*
Heptanonitrile	0.19	<0.05*	0.54	1.00
4-Methylthiazole	0.44	0.13	0.21	0.87
Octan-1-ol	0.14	0.07	0.75	0.38
4-(Methylthio)-butanenitrile	0.97	0.99	<0.05*	0.83
Nonanal	0.60	0.69	0.56	0.44
5-(Methylthio)-pentanenitrile	<0.05*	<0.05*	0.69	0.71
1,3-Bis(1,1-dimethylethyl)-benzene	<0.05*	0.09	0.54	<0.05*
Total aroma	0.49	0.62	0.27	<0.05*

For each Y-variable (O_2_, CO_2_, and storage time), the p-values from the Jack-knifing are presented. If p < 0.05 (indicated by *), the aroma compound has a significant effect on the model variable.

## Discussion

Traditional, round cabbage varieties have a good storability in cold conditions with high relative humidity. The pointed varieties, however, appear to suffer more due to the morphological differences in the structure of their heads (Gajewski et al., 2015). Some innovative Dutch growers currently obtain positive results to prolong storage life using CA, however, studies on these conical varieties and their storability are limited.

When produce is stored, weight loss occurs mainly due to both transpiration and respiration. This study found a mass loss (Figure 1) of 8% 94 dah and 18% 163 dah in normal air. This was significantly higher compared to the mass losses of 2.5% for cabbages heads stored in CA conditions. East et al. (2009) states that the main weight loss can be designated to water loss as result of the transpiration, while only a small part is due to the carbon loss through gas exchange. Decrease of O_2_ and increase of CO_2_ will reduce the respiration rate by inhibition of the decarboxylation reactions (Kader and Saltveit, 2002). To study the contribution of gas exchange to mass loss properly, the relative humidity in the different storage conditions should be kept similar. This could suggest that the differences measured in this study between the reference treatment and CA could be the effect of the difference in air circulation regardless of the storage conditions as in the reference method, the relative humidity is typically lower and a higher amount of ventilation is obtained compared to the closed system for CA treatments. Our results, however, are consistent with the results of studies that do keep the relative humidity of the different conditions similar. Gajewski et al. (2015) noted weight losses of, respectively, 6 and 7% for conical cabbages stored up to 3 months in 3 kPa O_2_ and 5 kPa CO_2_ or normal air. For the round varieties, Menniti et al. (1997) found weight losses >11% after 63 days of storage in normal air compared to <1% in CA, Osher et al. (2018) noted weight losses of 2–6% compared to 8% for cabbages stored for 90 days in CA and normal air, respectively. Choi et al. (2020) reported weight losses of 2.8 and 16.5% for winter kimchi cabbage heads stored in 2 kPa O_2_ and 5 kPa CO_2_ and normal air, respectively. This suggests that long-term storage of cabbage heads in CA reduces respiration and transpiration rates, therefore decreasing weight loss.

Besides the natural weight loss, an additional loss due to trimming off of yellowing, wilting and possible infected leaves should be considered. Although all CA conditions showed mostly green, healthy leaves with a limited amount of yellowing, while the leaves of cabbages stored in normal air all showed (partial) yellowing (Figure 2), a weight loss of approximately 20% was noted for all treatments 94 dah in order to obtain a product suited for marketing. These results are consistent with the results found by Gajewski et al. (2015) for cv Caraflex cabbages stored for 3 months in both normal air and CA. It should be mentioned that this trimming loss can be reduced by proper cultivar selection. The pointed cultivar ‘Bejo 2654’ showed a significant reduced trimming loss when stored in CA of <12% (Wang, 2002; Gajewski et al., 2015). At 163 dah, all CA treatments showed (partial) yellowing, while the leaves of the reference treatment were rotting and brown. The CA stored produce needed a more excessive amount of trimming (up to 26%), but were all still suited for commercialization. For the cabbages of the control group, however, the inner leaves showed yellowing and decay as well, meaning no amount of trimming was sufficient to obtain a good, marketable product. Our results were therefore congruous with studies on other cabbages types, stating that CA can extend storage life for several months (Menniti et al., 1997; Wang, 2002; Choi et al., 2020).

Although the consumers preference might slightly differ between cultures, usually, bright green colors are associated with a higher freshness. This green color is the result of the presence of chlorophyll in the leaves. Osher et al. (2018) found that cabbages stored in 2 kPa O_2_ and 5 kPa CO_2_ have higher chlorophyll levels compared to cabbages stored in normal air. Both Menniti et al. (1997) and Gajewski et al. (2015) reported a better color maintenance for CA treatments. In this study, the leaf scoring (Figure 3) showed similar results. All CA regimes maintained green, healthy leaves 94 dah on the day of opening the container. After a week at 7°C, yellowing of the leaves became more prominent. At 163 dah all CA-stored cabbages showed (partial) yellowing at SL0 and in some cases even brown spots at SL7. Cabbages stored in normal air showed a faster decay, with more prominent yellowing 94 dah and brown, rotten leaves appearing 163 dah. While this color evolution was significantly influenced by time and shelf life, we found that decreased levels of O_2_ and increased levels of CO_2_ significantly prolong color maintenance of the outer leaves (Table 1). The low O_2_ and high CO_2_ levels will result in a reduced ethylene synthesis and thus delayed chlorophyll degradation in the non-climacteric vegetative tissue of cabbage (Beaudry, 1999). Also, the increased levels of CO_2_ suppress the decay process by the blockage of the production of phenolic compounds and the inhibition of polyphenol oxidase (Siriphanich and Kader, 1985).

In addition to the outer appearance, internal quality is an important factor. When the cabbage head is cut in half from top to stem, discoloration can occur between the veins. Choi et al. (2020) reported that significantly less cabbages showed internal disorders or decay after 150 days of storage under 2 kPa O_2_ and 5 kPa CO_2_ (12.4–17.2%) compared to normal air (30%). Osher et al. (2018) described the positive effect of CA on development of gray rib in cabbage heads. In our study, however, no statistical differences were found based on the internal color measurements around the main vein (Table 2). For the SSC, different studies reported a slower sugar consumption in CA treatments (Raghavan et al., 1984; Suojala, 2003). Able et al. (2005) described the crucial role of sugar degradation in the regulation of the yellowing in detached pak choy leaves, with chlorophyll degradation only occurring after approximately 60% of the soluble sugars are utilized. If CA storage is able to delay sugar consumption, it will therefore positively affect both internal and external quality of the cabbages since sugar first gradually declines in the head tissues while accumulating in the core (Suojala, 2003). In our study a stable SSC was measured over time, apart from a small increase for cabbages stored in normal air (Table 2). The observed differences are too small to be noticeable for consumption. While the cabbages 163 dah stored in normal air where not marketable due to need of excessive trimming losing the expected tight, fresh pointed head, the core was not showing discoloration which could be due to the maintained SSC.

Measurements of the volatiles in the headspace surrounding the produce, showed that the aroma profile of cabbages changed over time. Seven aroma compounds were found to contribute considerably to the model describing the time effect (Table 3 and Supplementary Figure 3). The model, however, was not able to describe the effect of either O_2_ or CO_2_. From the Jack-knifing, it was found that formaldehyde, dimethyl disulfide and furan-2-ylmethanol could be good parameters to describe differences due to the gaseous conditions (Table 3 and Supplementary Figures 4, 5). Some of these compounds could be interesting volatile markers that might help determine the state of the cabbage within the cooling room without need for opening the containers. While acetone is a volatile with a pungent, characteristic odor (Burdock, 2010) and a well-known volatile marker as a rotting process indicator, it was found in fresh cabbages at harvest suggesting some fermentation and metabolic processes were already ongoing. A possible explanation could be the rather late harvest date. Ethyl acetate is a good fermentative marker (Jahromi et al., 2019), with an ethereal fruity flavor, somewhat nauseating in high concentrations (Burdock, 2010). Dimethyl disulfide is a predominant volatile compound in cabbage that contributes to the off-odor and is derived from *S*-methylcysteine sulfoxide (Akpolat and Barringer, 2015; Rajkumar et al., 2017). Dimethyl disulfide showed no significant change with time, O_2_, or CO_2_ separately, but is an important parameter since it shows a significant effect in the model for all the *Y* variables. It can be expected that it will increase when oxygen levels drop to a level resulting in fermentation, as reported by Menniti et al. (1997) and Dan et al. (1999) indicating increased production of off-flavors, such as dimethyl disulfide when oxygen levels drop to 1 kPa O_2_. However, when O_2_ levels are high, it might increase to, due to high metabolic activity. Kaji et al. (1993) states that increased CO_2_ helps to maintain cabbages in a good condition. From this study we can only conclude that the aroma profile of pointed cabbage changed over time regardless of the storage regime. In order to obtain a more robuust model that can predict the influence of the storage conditions, measurements need to be repeated with a higher number of repetitions for different O_2_ and CO_2_ levels.

*Brassica* vegetables are known to have high concentrations of sulfur-containing glucosinolates. The content and profile of these glucosinolates are susceptible to different environmental conditions, varieties, growing season, agricultural practices, and postharvest conditions (Bhandari et al., 2015; Osher et al., 2018). When the produce is damaged or – as in this study – is turned into juice, the enzyme myrosinase and the glucosinolates come together resulting in the hydrolysis of the glucosinolates into isothiocyanates, thiocyanates, and nitriles (Verkerk et al., 2001). In our study, several of such hydrolysis products are found to be influenced by the different *Y* variables. In this study, storage time and shelf life appeared to be the main factors to define the aroma profile of cabbage juice. A clear shift in aroma profile with an increasing storage duration (factor 1, Figure 7) and increasing shelf life (factor 3, Figure 9) is present. Whilst samples at harvest were clearly grouped together, the samples from the middle and the end of the season were less clearly separated. The shift in aroma profile was more outspoken for cabbages stored in normal air. The green, fresh volatiles that were highly correlated to the juices retrieved from fresh, green cabbages at harvest were mainly aldehydes and alcohols such as 1-penten-1-ol and 2-hexenal which was also found in other reports (Burdock, 2010; Radovich, 2010). Juices retrieved later in the storage season were characterized by high levels of nitriles (e.g., 2-butenenitrile, pentanenitrile) and sulfides (e.g., carbon disulfide). Although some try to enhance levels of glucosinolates in order to improve health, it is important to control it as it results in a bitter taste (Drewnowski and Gomez-Carneros, 2000). For marketing, it is important to note that the glucosinolate content is mostly influenced by time and temperature (Mérillon and Ramawat, 2017). In this study, also the level of O_2_ and CO_2_ showed to contribute to the model (Supplementary Figure 6). However, no clear relations were found. Increased levels of carbon disulfide, an odor with a pungent smell, were found for increased levels of both O_2_ and CO_2_. Green compounds such 3-hexenal and 1-penten-3-ol, showed no significant effect. However, 2-hexenal showed a decrease in CA. Different hydrolysis products of the glucosinolates such as 2-butenenitrile and 5-(methylthio)-pentanenitrile, showed, respectively, increased or decreased levels for CA stored cabbages (Supplementary Figures 9, 10). To study the effect of the gaseous conditions properly, more research is needed.

From this study, it is not possible to find a clear relation between the aroma measurements of the surrounding headspace and the cabbage juices. Both show a change in aroma profile over time, with a more prominent shift for cabbages stored in normal air. However, due to a limited amount of headspace measurements, no robuust model could be obtained describing the effect of O_2_ and CO_2_ on the aroma of the headspace surrounding the stored produce. Whilst quality measurements showed the benefits from CA storage, extending storage life to least 6 months, it is difficult to describe the effect of CA on the internal and external aroma of the cabbages.

## Conclusion

From this study we can conclude that storage under CA enables to store pointed cabbage for at least 6 months. Compared to normal air, pointed cabbages stored in CA maintained a better quality, i.e., better leaf scoring and less weight loss. In this study, the best results were found for the CA treatments with the lowest O_2_ level (1 kPa O_2_) and increased CO_2_ level (5 kPa CO_2_). The reduced O_2_ resulted in a better quality retention. Increased levels of CO_2_ delay browning and off-odors. The aroma profile of both the headspace surrounding the cabbages and the cabbage juices changed during CA storage. For the cabbage juices, there was also an effect of O_2_ and CO_2_. Cabbages stored in normal air were clearly different from cabbages stored in CA. Aroma may be an appropriate attribute to follow-up the metabolic state of the produce during storage, but more research is needed.

## Data Availability Statement

The original contributions presented in the study are included in the article/Supplementary Material, further inquiries can be directed to the corresponding author.

## Author Contributions

MJ, BV, MH, and BN contributed to the conception and design of the study. MJ performed the experimental work, data analysis, and wrote the first draft of the manuscript. BV contributed to statistical analysis of the quality data. MH contributed to the multivariate statistical analysis of the aroma data. All authors contributed to the manuscript revision, read, and approved the submitted version.

## Conflict of Interest

The authors declare that the research was conducted in the absence of any commercial or financial relationships that could be construed as a potential conflict of interest.

## Publisher’s Note

All claims expressed in this article are solely those of the authors and do not necessarily represent those of their affiliated organizations, or those of the publisher, the editors and the reviewers. Any product that may be evaluated in this article, or claim that may be made by its manufacturer, is not guaranteed or endorsed by the publisher.
