# Supplementary figures and images for: Quality Evolution and Aroma Profile of Pointed Cabbage in Different Storage Regimes
Source: Front Plant Sci. 2022 Apr 15;13:852817. doi: 10.3389/fpls.2022.852817 (PMC9051525; doi:10.3389/fpls.2022.852817)

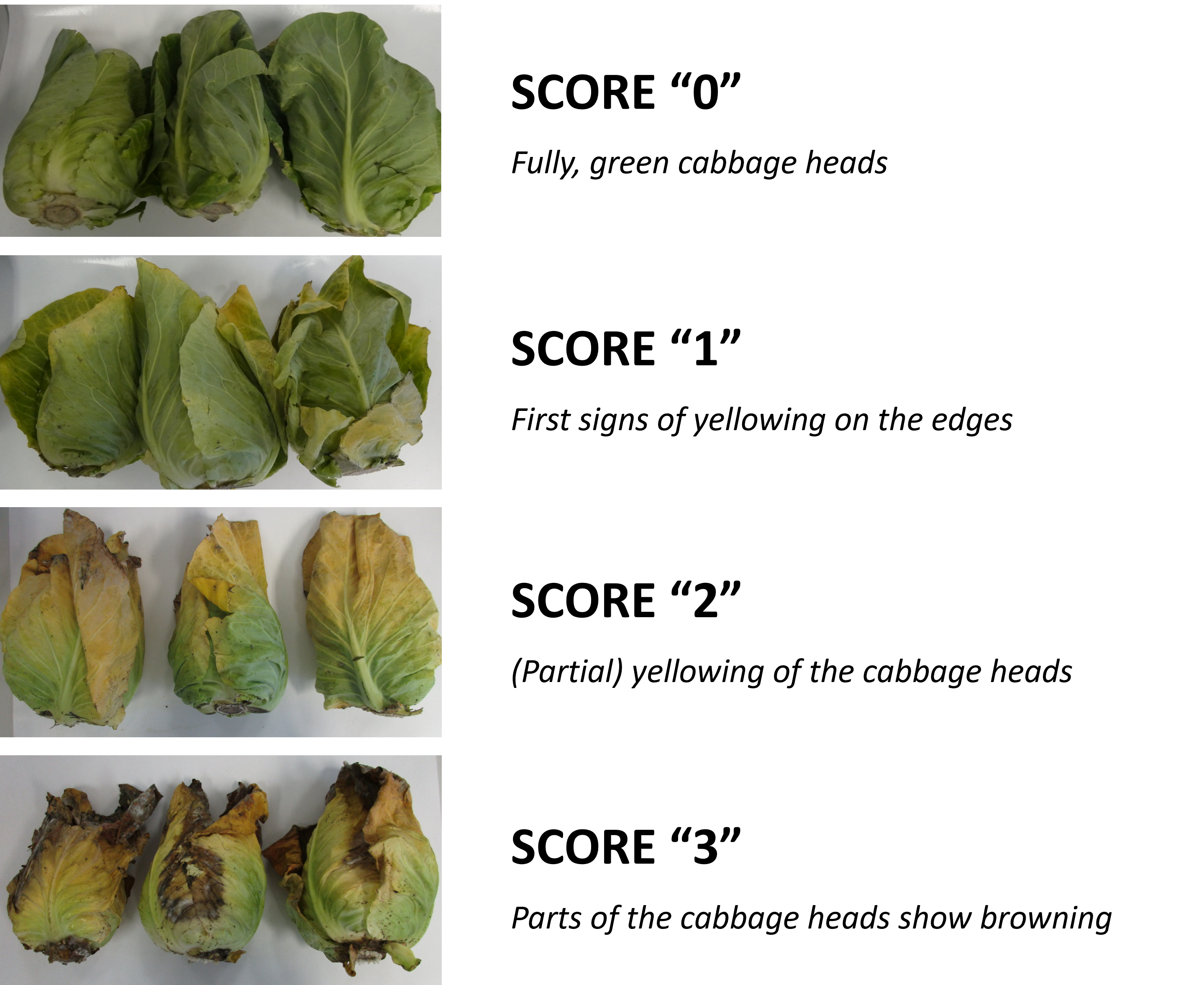

Supplement: Supplementary file 3 [file Image_1.PNG]
